# Identification and Analysis of Natural Killer Cells in Murine Nasal Passages

**DOI:** 10.1371/journal.pone.0142920

**Published:** 2015-11-17

**Authors:** Kazunari Okada, Shintaro Sato, Ayuko Sato, Ofer Mandelboim, Tatsuya Yamasoba, Hiroshi Kiyono

**Affiliations:** 1 Division of Mucosal Immunology, Department of Microbiology and Immunology, The Institute of Medical Science, The University of Tokyo, Tokyo, Japan; 2 Department of Otorhinolaryngology - Head and Neck Surgery, The Graduate School of Medicine, The University of Tokyo, Tokyo, Japan; 3 Core Research for Evolutional Science and Technology (CREST), Japan Science and Technology Agency, Tokyo, Japan; 4 The Lautenberg Center for General and Tumor Immunology, The Hebrew University Hadassah Medical School, Jerusalem, Israel; Karolinska Institutet, SWEDEN

## Abstract

**Background:**

Natural killer (NK) cells in the upper respiratory airways are not well characterized. In the current study, we sought to characterize and functionally assess murine nasal NK cells.

**Methods:**

Using immunohistochemistry and flow cytometry, we compared the nasal NK cells of *Ncr1* ^*GFP/+*^ knock-in mice, whose NK cells produced green fluorescent protein, with their splenic and pulmonary counterparts. In addition, we functionally analyzed the nasal NK cells of these mice *in vitro*. To assess the *in vivo* functions of nasal NK cells, C57BL/6 mice depleted of NK cells after treatment with PK136 antibody were nasally infected with influenza virus PR8.

**Results:**

Immunohistochemical analysis confirmed the presence of NK cells in the lamina propria of nasal mucosa, and flow cytometry showed that these cells were of NK cell lineage. The expression patterns of Ly49 receptor, CD11b/CD27, CD62L and CD69 revealed that nasal NK cells had an immature and activated phenotype compared with that of their splenic and pulmonary counterparts. Effector functions including degranulation and IFN(interferon)-γ production after *in vitro* stimulation with phorbol 12-myristate-13-acetate plus ionomycin or IL(interleukin)-12 plus IL-18 were dampened in nasal NK cells, and the depletion of NK cells led to an increased influenza virus titer in nasal passages.

**Conclusions:**

The NK cells of the murine nasal passage belong to the conventional NK cell linage and characteristically demonstrate an immature and activated phenotype. Despite their hyporesponsiveness *in vitro*, nasal NK cells play important roles in the host defense against nasal influenza virus infection.

## Introduction

Natural killer (NK) cells play important roles in host immune defense and have anti-tumor activity: they produce cytotoxic granules containing molecules such as perforins and granzymes to lyse infected or neoplastic cells [1]. Other important features of NK cells are their involvement in innate immune responses, including the production of cytokines including interferons (IFNs), their enhancement of local immune responses by directly acting on target cells, and their roles in the recruitment and activation of immune cells, including T cells and macrophages [1].

NK cells were previously considered to be a uniform population of cells with cytotoxic and cytokine-producing activities [1]. However, recent evidence regarding the surface marker expression and functional properties of NK cells has revealed their heterogeneity [2]. For example, the expression of CD27 and CD11b can be used to discriminate murine NK cells according to their maturation level [3, 4]. For example, CD11c^low^B220^+^ IFN-producing cells were originally considered to be killer dendritic cells but are now known to be activated NK cells that can produce various IFNs [4, 5]. These and similar investigations of mucosal NK cells helped to establish the concept of innate lymphoid cells (ILCs) [6].

ILCs, including NK cells, lack specific antigen receptors but can produce several effector cytokines (e.g., IFN-γ and interleukin(IL)-13), in response to stimuli such as infection and tissue damage [6]. In addition, ILCs contribute to the induction of lymphoid tissue organogenesis, homeostasis, and tissue repair [6]. For example, a subset of ILCs found in the murine small intestine and NK cells share expression of the surface marker NKp46; however, this intestinal ILC subset lacks the expression of other NK markers, such as NK1.1 and CD49b, and depends on the transcriptional factor RORγt for its development and differentiation [7, 8]. Furthermore, these RORγt^+^ ILCs are functionally unique in that they produce IL-22 [7–9]. Because IL-22 acts on mucous membranes and enhances mucosal barrier function [10], these so-called “ILC-22” cells play a key role in host immune defense.

Although some information regarding the NK cells and ILCs in the upper respiratory airways is available [11], they have not been well characterized. To address this deficit, we used *Ncr1* ^*GFP/+*^ knock-in mice [12], in which the NK-cell–specific marker *Ncr1* is replaced by green fluorescent protein (GFP), to confirm the presence of NK cells in the upper respiratory tract (i.e., nasal passages) and to analyze the immunologically and functionally unique characteristics of nasal NK cells, including their role in the clearance of nasally inoculated influenza virus.

## Materials and Methods

### Mice

C57BL/6 mice were purchased from Japan SLC (Shizuoka, Japan). ICR^nu/nu^ mice were purchased from Charles River Laboratories JAPAN (Kangawa, Japan). *Ncr1* ^*GFP/+*^ mice were generated as previously described [12] and housed under specific-pathogen–free conditions at the animal facility of the Institute of Medical Science, the University of Tokyo. Animal experiments were approved by and conducted in accordance with the guidelines of the Animal Care and Use Committee of the University of Tokyo. Mice were evaluated daily or every other day and remained clinically healthy during experiments, even after influenza viral infection. No mouse died due to experimental manipulation.

### Immunohistochemistry

Head tissues of 8-week-old *Ncr1* ^*GFP/+*^ mice were obtained after decapitation, fixed in 4% paraformaldehyde overnight at 4°C, preserved in 15% sucrose, and embedded in O.C.T. compound (Sakura Finetek, Tokyo, Japan); 6-mm sections of frozen nasal tissues were obtained [13]. Purified anti-GFP (A11122; Life Technologies, Carlsbad, CA, USA) and phycoerythrin–anti-mouse CD45 (30-F11; BD Biosciences, San Jose, CA, USA) were used as primary antibodies; biotinylated anti-rabbit IgG was used as the secondary antibody for anti-GFP and was detected by using the Cyanine 5 Tyramide Signal Amplification kit (NEL704A001KT or NEL705A001KT; PerkinElmer Life Sciences, Waltham, MA, USA). Sections were counterstained with 4′,6-diamidino-2-phenylindole (Sigma–Aldrich, St. Louis, MO, USA) and analyzed under a fluorescence microscope (BZ-9000, Keyence, Osaka, Japan).

### Cell preparation and flow cytometry

Splenic tissues were passed through a 70-μm mesh filter to obtain lymphocytes. Nasal and lung tissues were dissociated mechanically, and then treated twice by using RPMI1640 (Nacalai Tesque, Kyoto, Japan) supplemented with 0.5 mg/mL collagenase type IV (Wako Pure Chemical, Osaka, Japan) for 20 min with vigorous stirring at 37°C. Small intestine was treated by using RPMI1640 supplemented with 0.5 mM ethylenediaminetetraacetic acid, followed by RMPI1640 only, and then by RPMI1640 supplemented with collagenase with vigorous stirring at 37°C for 20 min each treatment. Collected cells were then enriched by using the Percoll (GE Healthcare, Little Chalfont, UK) gradient method [14]. Cells were stained with the appropriate fluorescence-conjugated antibodies. Anti-CD3 (clone, 145-2C11), anti-CD11b (M1/70), anti-CD27 (LG.3A10), anti-CD45 (30-F11), anti-CD49b (DX5), anti-CD69 (H1.2F3), anti-CD103 (R35-95), anti-CD107a (1D4B), anti-NK1.1 (PK136), and anti-IFN-γ (XMG1.2) antibodies were purchased from BD Biosciences; anti-Ly49A (A1), anti-Ly49C/F/H/I (14B11), anti-Ly49D (eBio4E5), anti-CD62L (MEL-14), anti-granzyme B (NGZB), and anti-2B4 (eBio24F4) were from eBiosciences (San Diego, CA, USA). We also used isotype-matched fluorescent-conjugated antibodies for control staining. Stained cells were evaluated by flow cytometry (FACS Canto II, BD Biosciences), and data were analyzed by using FlowJo software (Tree Star, Ashland, OR, USA).

### Cell stimulation and staining of granzyme B, CD107a, and intracellular IFN-γ

Mononuclear cells isolated from tissues (1 × 10^6^ cells/mL) were stimulated with phorbol 12-myristate-13-acetate (PMA) (200 ng/mL) and ionomycin (1 μg/mL) (Sigma) or with mouse IL-12 (20 ng/mL; R&D Systems, Minneapolis, MN, USA) and mouse IL-18 (5 ng/mL; Medical & Biological Laboratories, Nagoya, Aichi, Japan) for 4 h at 37°C in the presence of Golgistop (BD Biosciences). During the stimulation period, anti-CD107a antibody (5 μg/mL) or an isotype-matched control was added. After stimulation, intracellular IFN-γ was detected by using a Cytofix/Cytoperm Plus Fixation–Permeabilization Kit (BD Biosciences), followed by flow cytometric analysis. Granzyme B was detected as described for IFN-γ but without prior stimulation of the cells.

### Influenza virus infection

Influenza A/Puerto Rico/8/34 (H1N1, PR8; Sankyo Labo Service, Tokyo, Japan) was used for infection. Mice were infected intranasally with 10^3^ plaque-forming units (pfu) of PR8 diluted in 10 μL PBS (5 μL in each nostril) without anesthesia; this technique initially limits the infection to the nasal passages, and infection spreads to lung after several days [15]. On days 2 and 5 after infection, samples of nasal tissue were obtained for the analysis of cells by flow cytometry.

#### NK1.1^+^ cell depletion and titration of influenza virus in nasal passage

PK136 anti-NK1.1 antibody was purified from the ascites of ICR^nu/nu^ mice injected intraperitoneally with PK136 hybridoma cells (HB-191, ATCC, Vienna, VA, USA). To deplete NK1.1^+^ cells, each mouse (C57BL/6) was injected intraperitoneally with 100 μg of PK136 antibody (in 100 μL PBS) or PBS only (vehicle control). PK136 antibody was given on days –2, –1, and 2 relative to influenza inoculation (day 0). On days 2 and 5 after virus infection, whole nasal tissue was individually homogenized in 1 mL PBS by using a Tissue Lyser II (Qiagen, Venlo, The Netherlands). The resulting homogenates were examined by plaque-forming assay on Madin–Darby Canine Kidney (MDCK) cells as previously described [16]. Briefly, near-confluent 3.8-cm^2^ MDCK cell monolayers were infected with 100 μL of 10- to 10^4^-fold diluted aliquots of homogenate for 1 h at 37°C. These MDCK cells were washed with DMEM and then overlaid with 1 mL DMEM containing 1 mg/mL trypsin (GIBCO, Grand Island, NY, USA) and 1% agarose. Cultures were incubated at 37°C with 5% CO_2_ for 72 h, after which plaques were visualized by using crystal violet.

### Statistical analyses

To analyze data, the Mann–Whitney *U* test was used; Ryan’s multiple-comparison method was included as needed. Significance was defined as a *P* value of <0.05.

## Results

### Visualization of nasal NKp46^+^ cells

**Fig 1:**
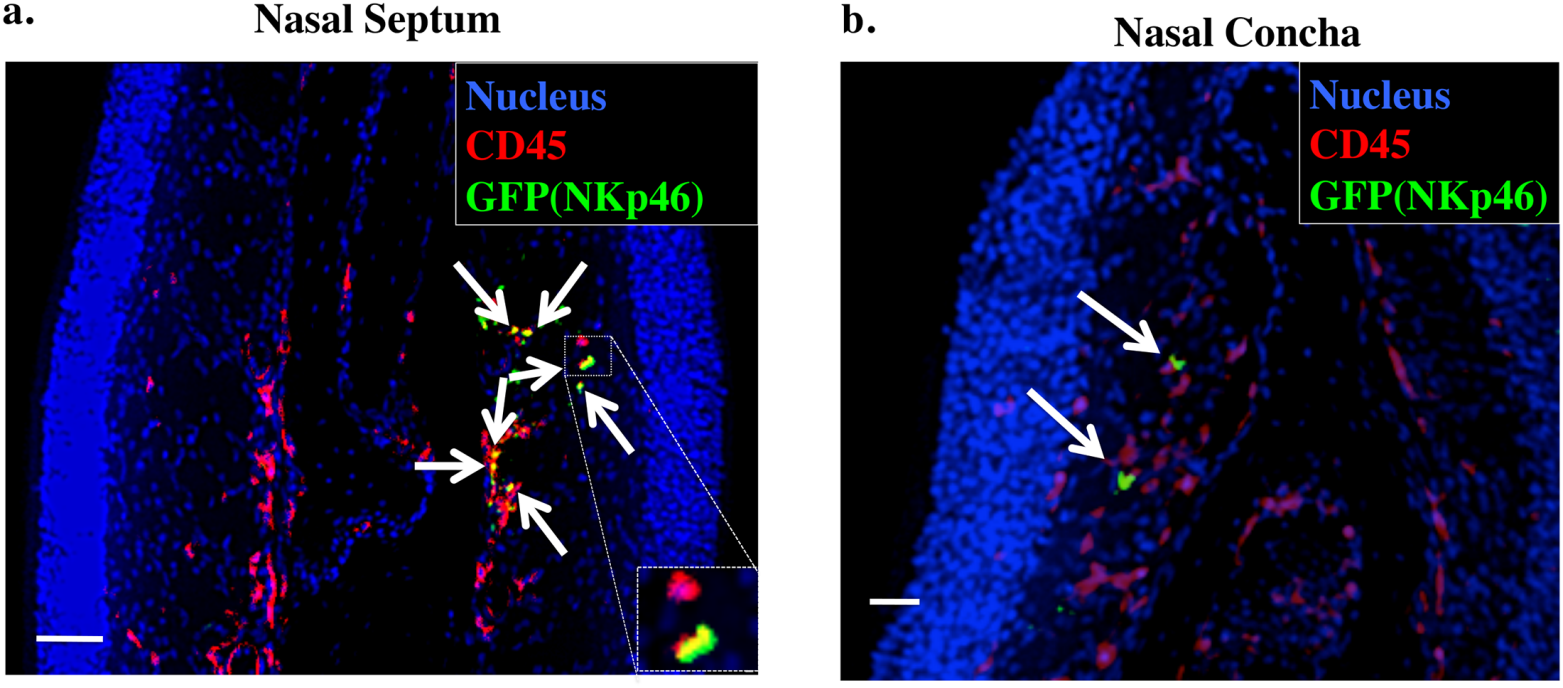
Visualization of nasal NKp46^+^ cells by using immunohistochemistry. Frozen sections of nasal tissue obtained from 8-week-old *Ncr1* ^*GFP/+*^ mouse were stained with 4′,6-diamidino-2-phenylindole (nucleus), anti-CD45, and anti-GFP antibodies and examined under a fluorescence microscope. Arrows indicate CD45^+^GFP(NKp46)^+^ cells. Bar, 50 μm. Data are representative of at least 3 independent experiments. a. Nasal septum. b. Nasal concha.

Murine NK cells express the natural cytotoxicity receptor NKp46, which is encoded by the *Ncr1* gene [17, 18]. To facilitate the identification of the possibly few NK cells present in mouse nasal passages, we used *Ncr1* ^*GFP/+*^ knock-in mice [8, 12]. Because this gene-manipulated strain of mice expresses a GFP reporter under the control of the *Ncr1* promoter, GFP^+^ cells are considered to be NK cells. As shown in Fig 1a, GFP^+^NKp46^+^ cells were located in the lamina propria region of nasal mucosa, similar to their location in the small intestinal lamina propria [7, 8]. In addition, GFP^+^NKp46^+^ cells were present in the nasal concha, albeit at a lower level than in the nasal passages (Fig 1b).

### Characteristics of nasal NKp46^+^ cells

**Fig 2:**
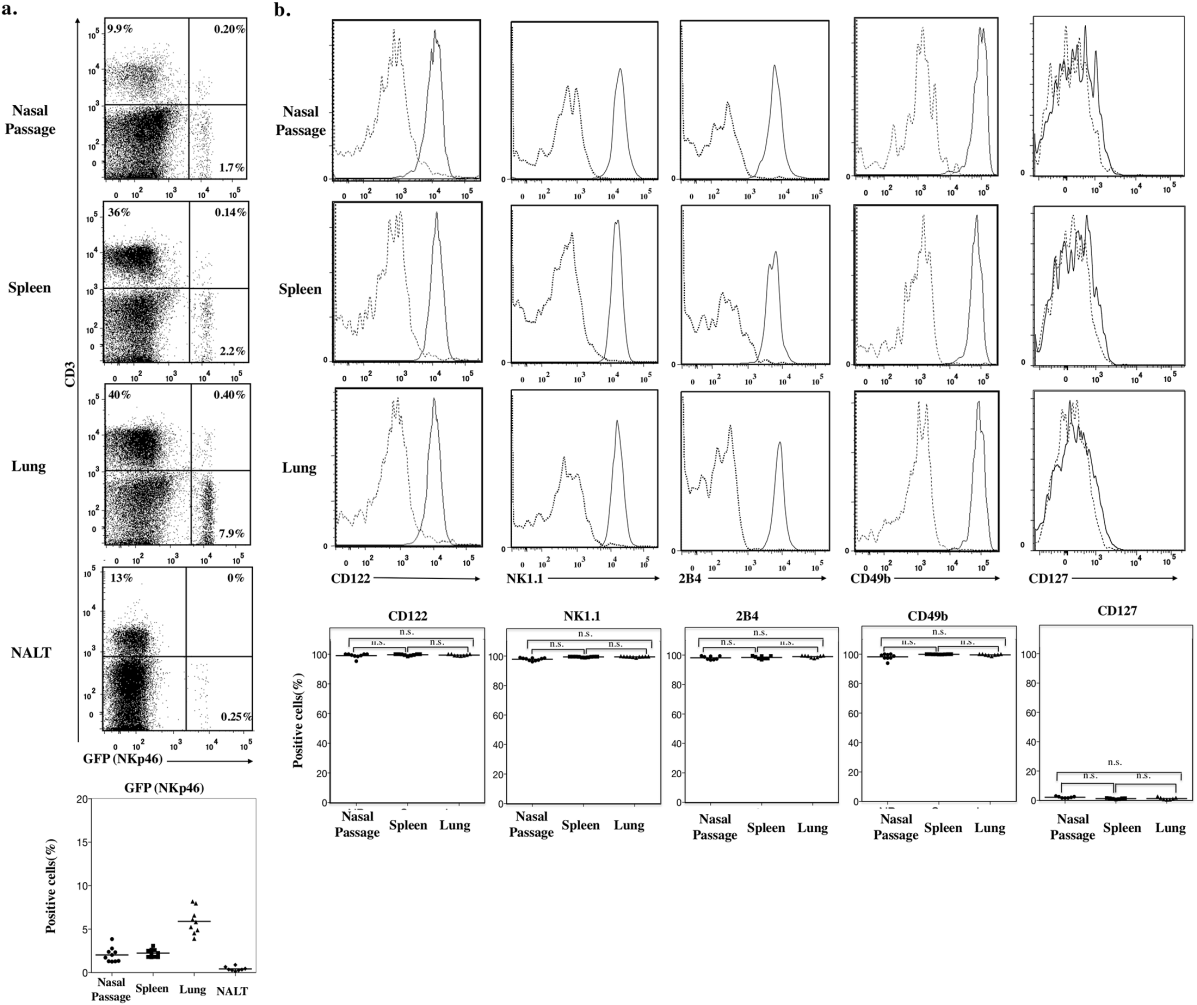
Nasal NKp46^+^ cells are NK-lineage cells. a. Flow cytometry of CD45^+^ cells from nasal passage, spleen, lung, and Nasopharynx associated lymphoid tissue (NALT) of *Ncr1* ^*GFP/+*^ mice stained with CD3. Numbers in quadrants indicate the percentages of cells in each. Dot plot below shows percentage of GFP (NKp46)^+^ cells in CD45^+^ cells from nasal passage, spleen, lung, and NALT. b. Flow cytometry of CD3^−^NKp46^+^ cells from spleen, lung, and nasal passages stained with CD122, NK1.1, 2B4, CD49b, and CD127. Continuous lines, specific antibodies; Dashed lines, isotype-matched control antibodies. Dot plot below shows the percentage of positively stained cells. Bar, mean; n.s.; not significant (Mann-Whitney *U* test with Ryan’s multiple comparison method). Data are obtained from at least 3 independent experiments.

To define the unique immunobiological characteristics of nasal NK cells, mononuclear cells from the nasal passages and nasopharyngeal associated lymphoid tissue of *Ncr1* ^*GFP/+*^ mice were analyzed for the expression of cell-surface molecules by using flow cytometry. For comparison, splenic and pulmonary NKp46^+^ cells from same mice were evaluated. CD3^−^NKp46^+^ cells accounted for approximately 1.7% of the CD45^+^ lymphocytes in nasal passages, with a similar frequency found in spleen (Fig 2a). In contrast, few CD3^−^NKp46^+^ cells (less than 0.5% of CD45^+^ lymphocytes) were found in nasopharyngeal associated lymphoid tissue (Fig 2a).

Conventional NK cells (included in group 1 ILCs) [6] express a variety of cell-surface markers including CD122 (IL-2Rβ) [19], CD161 (recognized by the NK1.1 antibody) [4], 2B4 receptor (which mediates non-major histocompatibility complex(MHC)-restricted cell killing), and CD49b (DX5; also known as adhesion molecule integrin α-2) [4, 20]. Here, nasal NKp46^+^ cells expressed these NK-specific surface molecules in the same patterns as did splenic and lung NK cells (Fig 2b). In addition, nasal NKp46^+^ cells did not express CD127 (IL-7Rα) (Fig 2b) whereas those from the lamina propria of the small intestine did express CD127 (S1 Fig). Our results suggest that nasal NKp46^+^ cells belong to the classic NK-cell lineage and are not RORγt^+^ ILCs, which are NK1.1^–^ and CD127^+^ [6]. Therefore, our results suggest that, like their splenic and pulmonary counterparts, nasal NKp46^+^ cells are NK cells.

### Expression of Ly49 family receptors in nasal NK cells

**Fig 3:**
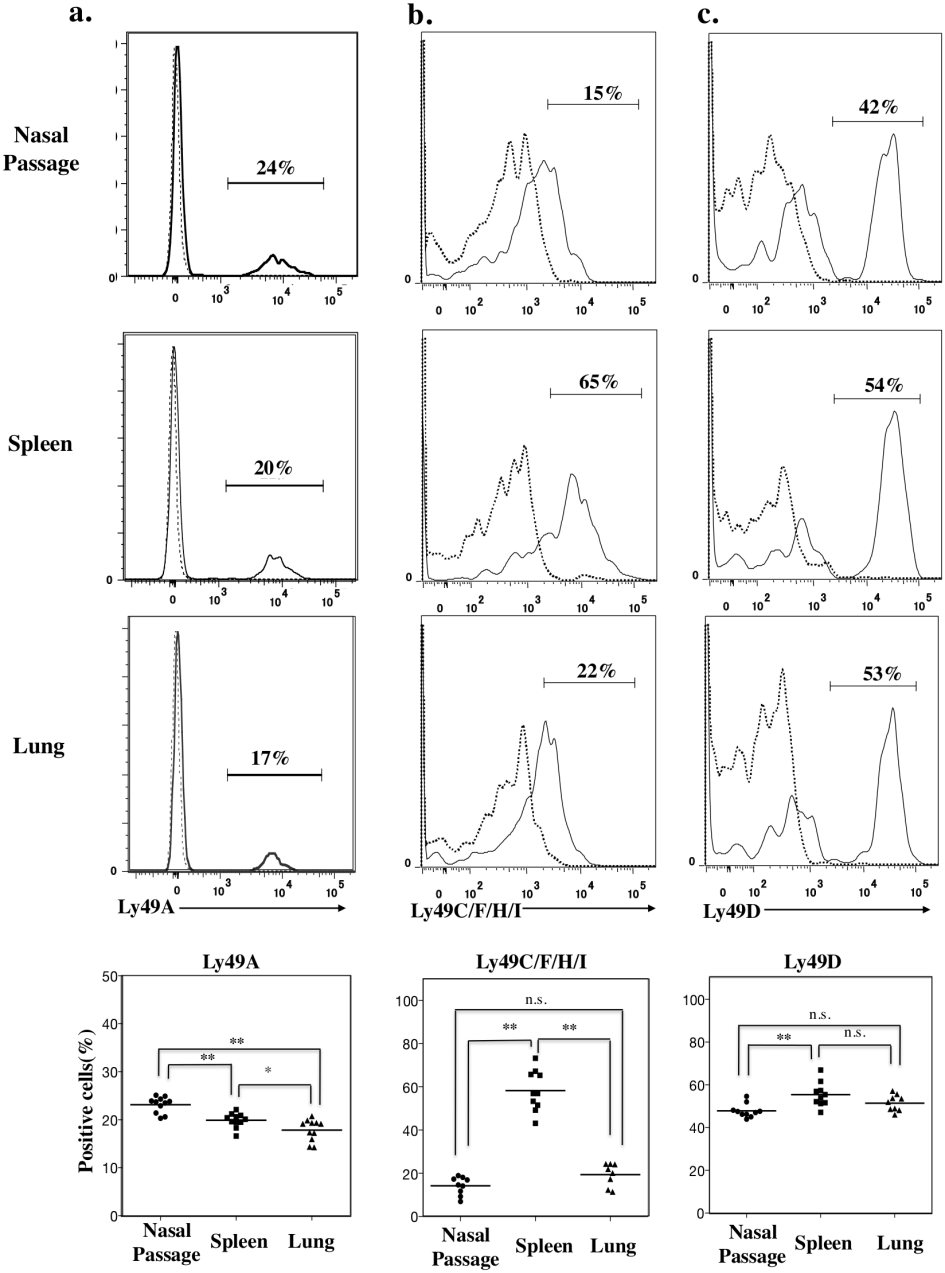
Nasal NK cells have a unique expression pattern of Ly49 family receptors. a, b, c. Flow cytometry of CD3^−^NKp46^+^ cells from nasal passage, spleen, and lung stained with (a) Ly49A, (b) Ly49C/F/H/I, and (c) Ly49D. Numbers in histograms indicate the percentages of positive cells. Continuous lines, specific antibodies; Dashed lines, isotype-matched control antibodies. Dot plots below shows the percentage of positively stained cells. Bar, mean; n.s., not significant; *, *P* < 0.05; **, *P* < 0.01 (Mann-Whitney *U* test with Ryan’s multiple comparison method). Data are obtained from at least 3 independent experiments.

The Ly49 receptor family consists of a cluster of transmembrane C-type lectin receptors [17, 21]. NK cells express various Ly49 receptors, which recognize MHC class Ia molecules expressed by target cells [17, 21]. Ly49 receptors transmit activating or inhibitory signals to regulate the cytotoxicity of NK cells against normal host cells [18]. Here, although NK cells from murine nasal passages, spleen, and lung all expressed a repertoire of Ly49 family receptors, the expression pattern differed depending on the site from which the NK cells were isolated (Fig 3). A slightly higher percentage of NK cells in nasal passage expressed Ly49A than did those in lung or spleen (Fig 3a) (*P* < 0.01, Mann–Whitney *U* test with Ryan’s multiple comparison method), and a greater percentage of splenic NK cells expressed Ly49C/F/H/I than did nasal and pulmonary NK cells (*P* < 0.01, Mann–Whitney *U* test with Ryan’s multiple comparison method) (Fig 3b). In addition, the percentage of Ly49D^+^ NK cells in nasal passages was slightly but reproducibly less than that in spleen and lung (*P* < 0.01, Mann–Whitney *U* test with Ryan’s multiple comparison method) (Fig 3c).

### Unique maturation and activation patterns of nasal NK cells

**Fig 4:**
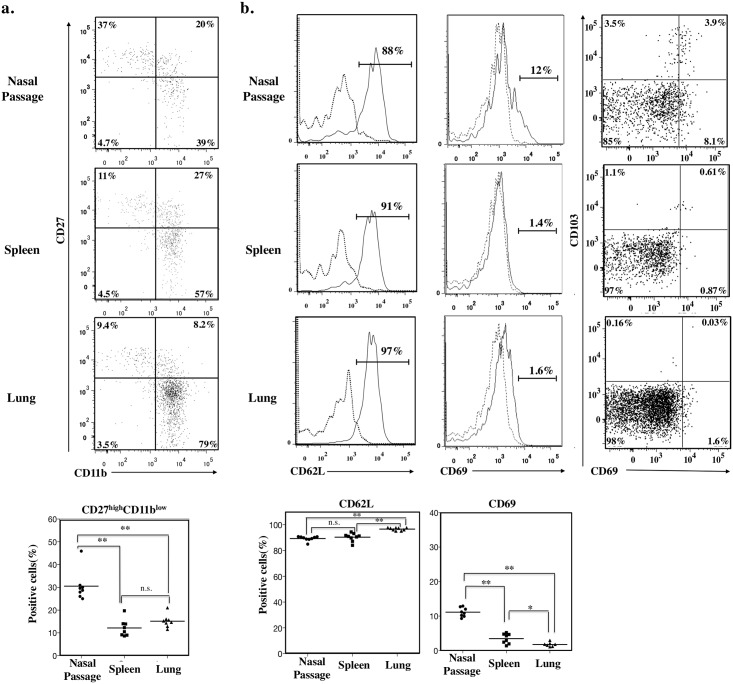
Unique maturation and activation status of nasal NK cells. a. Flow cytometry of CD3^−^NKp46^+^ cells from nasal passage, spleen, and lung, and double-stained with CD11b and CD27. Numbers in quadrants indicate the percentages of cells in each. Dot plots below shows percentage of CD27^high^CD11b^low^ cells from nasal passage, spleen, and lung. b. Flow cytometry of CD3^−^NKp46^+^ cells from spleen, lung, and nasal passages with CD62L, CD69, and CD69/CD103. The numbers in the histograms indicate the percentage of positive cells. Solid line, specific antibody; dashed line, isotype-matched control antibody. Dot plots below shows the percentage of positively stained cells. Bar, mean; n.s.; not significant; *, *P* < 0.05**, *P* < 0.01 (Mann-Whitney *U* test with Ryan’s multiple comparison method). Data are obtained from at least 3 independent experiments.

CD27 and CD11b molecules have been used to classify NK cells into four different developmental stages [22, 23]. We used these markers to analyze the maturation pattern of NK cells from mouse nasal passages. Consistent with a previous report [3], splenic NK cells included large populations of CD27^high^CD11b^high^ mature and CD27^low^CD11b^high^ senescent cells and few CD27^high^CD11b^low^ immature cells (Fig 4a). In comparison, most lung NK cells comprised a CD27^low^CD11b^high^ senescent population (Fig 4a), whereas nasal NK cells included numerous CD27^high^CD11b^low^ immature cells (*P* < 0.01, Mann–Whitney *U* test with Ryan’s multiple comparison method) (Fig 4a). Compared with those from lung and spleen, fewer nasal NK cells expressed CD62L (*P* < 0.01, Mann–Whitney *U* test with Ryan’s multiple comparison method; Fig 4b), the expression of which is known to be unregulated according to the maturation of NK cells [3].

In addition, nasal NK cells showed up-regulated expression of CD69 (*P* < 0.01, Mann–Whitney *U* test with Ryan’s multiple comparison method; Fig 4b), a known maturation and activation marker of NK cells [24, 25]. We then examined the NK-cell expression of CD69 and CD103, which are expressed by lymphocytes that reside in mucosal tissue [26]. About one-third of CD69^+^ nasal NK cells are CD103^+^ (Fig 4b), suggesting that the majority of nasal NK cells are activated and express CD69 independent of whether they reside in tissue.

### Effector functions of nasal NK cells

**Fig 5:**
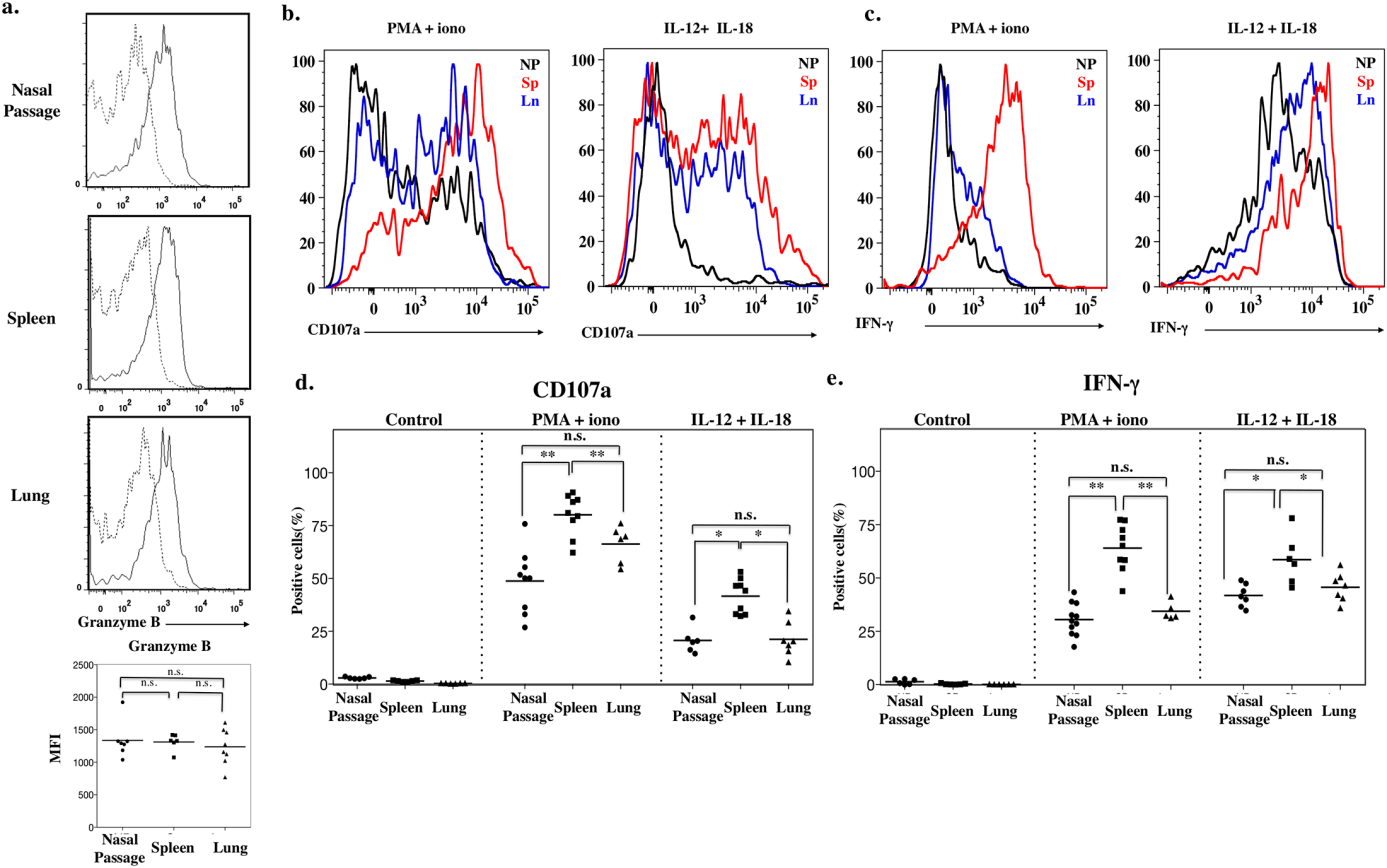
Impaired effector function of nasal NK cells. a. Intracellular expression of granzyme B by CD3^−^NKp46^+^ cells. Dashed line, isotype-matched control antibody; solid line, specific antibody. Dot plots below shows the mean fluorescence intensity (MFI). b, c. Intracellular staining of (b) CD107a and (c) IFN-γ production by lymphocytes isolated from nasal passage, spleen, and lung and stimulated for 4 h with various stimuli. Isolated lymphocytes were stimulated and then stained for surface antigen followed by intracellular staining. Signal assessed by gating on CD3^−^NKp46^+^ cells. Black line, nasal passage; red line, spleen; blue line, lung. d, e. Dot plots showing the percentage of positively stained cells after stimulation. Control, isotype matched control; PMA/iono, phorbol-12-myristate-13-acetate and ionomycin. Bar, mean; n.s.; not significant; *, *P* < 0.05 **, *P* < 0.01 (Mann-Whitney *U* test with Ryan’s multiple comparison method). Data are obtained from at least 3 independent experiments.

The results of our surface-molecule expression analysis of CD69 (Fig 4b) imply that a subset of nasal NK cells is in a functionally activated state. Because granzyme B is a serine protease and is a component of the cytoplasmic granules of NK cells, its expression directly correlates with the cytotoxicity of NK cells [27, 28]. The granzyme B level of nasal NK cells isolated from *Ncr1* ^*GFP/+*^ mice was very similar to those of splenic and pulmonary NK cells (*P* > 0.05, Mann–Whitney *U* test with Ryan’s multiple comparison method; Fig 5a).

CD107a, also known as lysosome-associated membrane protein 1 (LAMP-1) [29], is up-regulated on the surfaces of CD8^+^ T cells [30] and NK cells [31] after their stimulation, and its expression correlates well with the degranulation of chemical mediators and cytotoxic activity [31]. When stimulated with PMA and ionomycin, most splenic NK cells from *Ncr1* ^*GFP/+*^ mice expressed CD107a on their cell surfaces, consistent with the results of a previous study [31], indicating that these cells possess cytotoxic activity. Compared with splenic NK cells, pulmonary and nasal NK cells showed significantly less up-regulation of CD107a surface expression (*P* < 0.05, Mann–Whitney *U* test with Ryan’s multiple comparison method; Fig 5b and 5d). Similar trends were seen after the exposure of these various NK cell populations to IL-12 plus IL-18 (Fig 5c and 5e), in that the frequencies of nasal and pulmonary IFN-γ^+^ NK cells were significantly lower than that of splenic NK cells Mann-Whitney *U* test with Ryan’s multiple comparison method; Fig 5c). These results suggest that nasal NK cells are functionally hyposensitive to typical stimulation regimens that induce cytotoxic activation.

### Role of nasal NK cells in influenza infection

Although nasal NK cells showed appropriate levels of granzyme B expression during the resting (or naïve) state (Fig 5a), these cells seemed to have lower degranulation ability (Fig 5b) and to produce lower levels of IFN-γ after *in vitro* stimulation (Fig 5c). We next investigated whether nasal NK cells act as initial innate effector cells during *in vivo* influenza virus infection.

We adopted a commonly used influenza virus infection model [15, 32] coupled with or without depletion of NK cells by intraperitoneal injection of anti-NK1.1 antibody [33, 34]. Anti-NK1.1 antibody treatment successfully eliminated almost all splenic, pulmonary, and nasal NK cells by day 2 after injection (S2 Fig). Unanaesthetized mice were challenged intranasally with small amounts of virus-containing fluid (as little as 10 μL total, 5 μL in each nostril); this technique initially limits the infection to the nasal passages, and infection spreads to lung after several days [15].

**Fig 6:**
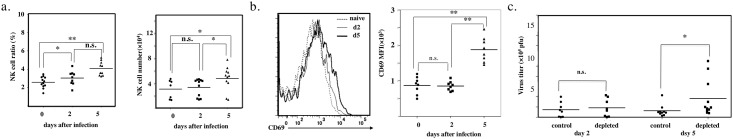
Indispensable role of nasal NK cells in influenza virus infection. a. Change of nasal NK cells after infection with PR8 1×10^3^ pfu/mouse intranasally. Horizontal axis, day after infection; vertical axis, percentage of nasal NK cells in CD45^+^ lymphocytes (left) or absolute count of nasal NK cells (right). *, *P* < 0.05 **, *P* < 0.01 (Mann-Whitney *U* test with Ryan’s multiple comparison method). Data are obtained from at least 3 independent experiments. b. CD69 expression of nasal NK cells after intranasal infection with influenza virus PR8 (1×10^3^ pfu/mouse). Histogram (left). Dashed line, naïve; solid line, day 2 after infection; bold line, day 5 after infection. Dot plot of MFI (right). Data are representative of 2 independent experiments with 4 mice. c. Nasal virus titer of mice intranasally infected with influenza virus PR8 (1×10^3^ pfu/mouse). Mice were injected intraperitoneally with 100 mg PK136 antibody or an isotype-matched control on days –2, 0, 2 after infection. Bar, mean; horizontal axis, day after infection; vertical axis, virus titer (pfu). Data are representative of 3 independent experiments with 4 to 6 mice in each group. n.s., not significant; *, *P* < 0.05 **, *P* < 0.01 (Mann-Whitney *U* test with Ryan’s multiple comparison method). Data are obtained from at least 3 independent experiments.

These mice, which had been inoculated with 1 × 10^3^ pfu influenza A virus PR8, were examined for changes in the nasal NK cell population over time. Despite their hyporesponsiveness (Fig 5b and 5c), nasal NK cells were increased significantly in percentage on day 2 (*P* < 0.05) and day 5 (*P* < 0.01), as well as in number on day 5 (*P* < 0.05), compared with those of day 0 after nasal challenge with PR8 (Mann–Whitney *U* test with Ryan’s multiple comparison method; Fig 6a). Furthermore, the level of CD69 expression by nasal NK cells was significantly higher on day 5 than on days 0 or 2 (*P* < 0.05, Mann–Whitney *U* test with Ryan’s multiple comparison method; Fig 6b).

We then compared viral growth in the nasal passage between the NK-cell–depleted and control groups. Whereas NK cell depletion had no effect on the amount of virus in the nasal passages during the immediate phase of the infection (e.g., day 2 after infection), viral titers on day 5 were significantly higher in the nasal passages of NK-cell–depleted mice compared with control mice (*P* < 0.05, Mann–Whitney *U* test; Fig 6c). These findings suggest that nasal NK cells respond to influenza infection and consequently inhibit virus replication, especially during the early stages of the infection.

## Discussion

We have demonstrated the presence of NK cells in murine nasal passages (upper respiratory tract) (Figs 1 and 2) and have shown that, compared with splenic and pulmonary NK cells, nasal NK cells have decreased expression of Ly49 family receptors and are relatively immature, but they can be activated under naïve conditions (Fig 3). Despite their up-regulated expression of activation markers such as CD69, nasal NK cells somehow respond less dramatically to stimuli such as plus and IL-12 plus IL-18, release fewer granules, and produce less IFN-γ than do their splenic counterparts (Fig 5d and 5e). Despite the hyporesponsiveness and relatively small number (no more than 10^4^ cells per mouse) of nasal NK cells, our current results suggest an indispensable role of nasal NK cells in the control of nasal influenza virus infection, during which they inhibit viral growth at the early stages of the infection (Fig 6c).

NK cells are distributed in various organs, including primary and secondary lymphoid tissues, such as the thymus and peripheral lymph nodes, respectively [35], non-lymphoid tissues such as liver [3, 36, 37] and skin [7], and mucosal tissues such as the reproductive [25], digestive [7, 8], and lower respiratory [3, 34] tracts. The phenotypes and functions of NK cells differ depending on the source organ or tissue. Because the nasal passages are a ‘front line’ in respiratory defense, the characteristics and functions of nasal NK cells should be clarified, but little information is currently available regarding NK cells in upper respiratory airways. Our current study revealed the unique characteristics of nasal NK cells.

To identify NK cells efficiently and precisely, we used *Ncr1* ^*GFP/+*^ knock-in mice, which serve as NK cell reporter mice [8, 12]. The immunohistochemical analysis confirmed the presence of NK cells in the lamina propria of nasal mucosa, as in the intestines and skin [7, 8]. In addition, we verified that nasal NKp46^+^ cells are CD3^−^ and belong to the conventional NK-cell (member of group 1 ILCs), rather than to the recently identified other ILC lineages (Fig 2a and 2b) [6]. Conventional NK cells express CD122, NK1.1, 2B4, and CD49b [4]. All of these markers are present on nasal NKp46^+^ cells (Fig 2b) and their splenic and pulmonary counterparts. Both conventional NK cells and RORγt^+^ ILCs are known to express NKp46 as well as CD127 [6], but unlike in the lamina propria of the small intestine [7, 8], RORγt^+^ ILCs were not present in nasal passages, because these tissue samples lacked NKp46^+^NK1.1^−^CD127^+^ cells (Fig 2b).

The Ly49 receptor family is important for discrimination between normal and neoplastic or infected cells [17, 38]. Each NK cell is thought to expresses one to three Ly49 receptor(s) stochastically. The precise mechanism regulating the expression of these receptors remains unclear [38], but a recent report indicates that the expression of Ly49 receptors, as well as other surface molecules, is regulated by pathogens such as influenza virus, *Staphylococcus aureus*, and *Klebsiella pneumonia* [38]. Here, we showed that the expression repertoire of Ly49 family receptors of NK cells differed slightly among nasal passage, spleen, and lung (Fig 3). Therefore, we speculate that differences in the microenvironments (including commensal bacteria) among these tissues may result in the diverse expression of Ly49 family receptors.

Like surface molecule expression, the maturation and activation patterns of NK cells varies among their anatomic sites [3, 9, 10], reflecting NK cell function *in situ*. In terms of maturation, CD27 and CD11b frequently are used to differentiate NK cells according to CD27^high^CD11b^low^ immature, CD27^high^CD11b^high^ mature, and CD27^low^CD11b^high^ senescent subpopulations [3]. Bone marrow and lymph node NK cells are CD27^high^, that is, relatively immature cells, whereas hepatic and blood NK cells tend to be CD11b^high^, or relatively mature cells [3]. We found that the nasal NK cell population has a unique CD27–CD11b pattern (Fig 4a), with a significant abundance of CD27^high^CD11b^low^ immature cells, whereas most splenic NK cells were CD11b^high^ mature cells and most pulmonary NK cells were CD27^low^CD11b^high^ senescent cells, as reported previously [3]. This speculation is supported by our finding that the percentage of NK cells expressing CD62L (a marker known to be upregulated as NK cells mature [3]) was lower in nasal passages than in lung of spleen (Fig 4b), given that CD62L is upregulated as NK cells mature [3]. We also studied other activation-related markers, such as CD69 [24], an adhesion molecule required for the migration of cells from the blood circulation to peripheral tissues. Because CD69 is also known as a marker of mucosal-tissue-resident cells, [26] we examined its co-expression with CD103, another molecule expressed by resident cells at mucosal sites (Fig 4b). Only one-third of CD69^+^ NK cells also expressed CD103, and we therefore speculate that an as-yet unknown factor induces CD69 expression, or spontaneous activation of nasal NK cells, regardless of mucosal residency.

Here, we have shown that splenic, pulmonary, and nasal NK cells contain nearly equal amounts of granzyme B in secretion granules (Fig 5a). However, CD107a expression levels indicate weaker degranulation activity of nasal NK cells in response to stimulation with PMA–ionomycin or IL-12–IL-18, compared with their splenic counterparts (Fig 4b). In addition, IFN-γ production by nasal NK cells is reduced in response to these stimuli (Fig 4c). Dermal NK cells share some features with nasal NK cells, such as elevated CD69 expression and hyporesponsiveness *in vitro* [7]. This resemblance suggests a possible interaction between NK cells and commensal bacteria, which shape various characteristics of NK cells, as does expression of Ly49 receptors[17]. The lower respiratory airway is relatively sterile under healthy conditions, and during infection, pulmonary cells need to respond promptly. It therefore is rational that fully mature, non-activated NK cells reside in the lung. In contrast, nasal passages [39], like the skin [40], are constantly stimulated by foreign antigens and pathogens, and to respond to these stimuli, nasal and dermal NK cells may acquire and maintain an activated phenotype. Studies of the interaction between nasal NK cells and bacteria, including both pathogenic and commensal organisms, likely would clarify the cause of the tissue-specific activation status of NK cells. Given their lack of NK-typical functions, nasal NK cells might have a unique function other than producing IFN-γ and cytotoxic granules.

The hyporesponsiveness of nasal NK cells prompted us to investigate their functional role in nasal infection *in vivo*. We hypothesized that NK cells would have a minor effect on decreasing infection. The importance of NK cells in controlling virus infections has long been under extensive discussion [33, 41, 42]. In the present study, we adapted a nasal influenza virus infection model [15, 32], because controlling nasal infection is supposed to be an important arm of the surface defense system for the prevention of airway infection and subsequent systemic progression. On day 2, CD69 expression was not upregulated in nasal NK cells, and the absence of NK cells did not change the amount of virus in the nasal passage (Fig 6b and 6c). From this result, we consider that NK cells in nasal passages are unresponsive to influenza viral infection before day 2. However, despite the hyporesponsiveness of nasal NK cells, NK cell depletion resulted in increased influenza viral titers in the nasal passages on day 5 after intranasal infection (Fig 6c). On day 5, we noted increases of both cell number and CD69 expression of nasal NK cells (Fig 6a and 6b). Therefore, between day 2 and day 5, the NK cells in the nasal passages apparently respond to influenza virus infection and inhibit viral replication. As for nasal NK cells, the depletion of pulmonary NK cells leads to an increase in influenza viral titer [31]. We were unable to determine whether the accumulation of nasal NK cells was due to the proliferation of local NK cells or to the migration of circulating NK cells, but the number of NK cells on day 5 was not more than twice that on day 2 (Fig 6a). Therefore, the majority of NK cells found in nasal passages on day 5 are probably local resident NK cells, and without these cells, nasal influenza virus infection is difficult to control.

Protection against viral infection is thought to be controlled through multilayered surface and systemic barrier systems [43]. In the proposed mechanism, innate immune cells such as NK cells and γδ T cells first initiate an innate immune response, which is supported by cytokines including IL-12 and IL-18 produced by antigen-presenting cells [43]. This process is followed by an acquired immune response, in which antibody-producing B cells and helper–cytotoxic T cells play an important role [43]. As shown in previous studies [44, 45], our results indicate the function of NK cells in the nasal innate immunity, against influenza virus during the early phases of infection. Similar to the pulmonary NK cells of aged mice [34], hyporesponsive nasal NK cells still contribute to the clearance of influenza virus, perhaps at the initial stage of upper respiratory infection. Although the ability of nasal NK cells to respond to extracellular stimuli is lower than those of splenic and pulmonary NK cells (Fig 5b and 5c), nasal NK cells can and do respond sufficiently to control influenza infection. Additional study of the activity of nasal NK cells against other viruses, such as rhinovirus and adenovirus, is important to gain a complete understanding of nasal NK-cell–mediated innate immunity.

## Conclusions

We used *Ncr1* ^*GFP/+*^ knock-in mice to confirm the presence and elucidate the phenotypes of immunologically important nasal NK cells. Compared with those of spleen and lung, nasal NK cells showed a unique maturation and activation status. Although nasal NK cells had an immature and activated phenotype, they responded less dramatically than did their lung counterparts to extracellular stimuli *in vitro*. Despite their hyporesponsiveness, nasal NK cells contributed to the control of nasal influenza virus infection *in vivo*.

## Supporting Information

S1 Fig Expression of CD127 by NKp46^+^ cells in lamina propria of short intestine. Flow cytometry of CD3^−^NKp46^+^ cells from lamina propria of short intestine stained with CD127. Data are representative of 4 mice. Continuous lines, specific antibodies; Dashed lines, isotype-matched control antibodies.
(TIF)

S2 Fig Depletion of NK1.1^+^ cells by using anti-NK1.1 antibody. Depletion of nasal, splenic, and pulmonary NK cells. Lymphocytes of *Ncr1* ^*GFP/+*^ mice were analyzed 2 days after intraperitoneal injection of (right) 100 μg PK136 antibody or (left) isotype-matched control antibody. Horizontal axis, GFP (NKp46); vertical axis, NK1.1. Data are representative of 2 independent experiments using 4 mice each.
(TIF)
