# Supplementary figures and images for: Identification and Analysis of Natural Killer Cells in Murine Nasal Passages
Source: PLoS One. 2015 Nov 17;10(11):e0142920. doi: 10.1371/journal.pone.0142920 (PMC4648567; doi:10.1371/journal.pone.0142920)

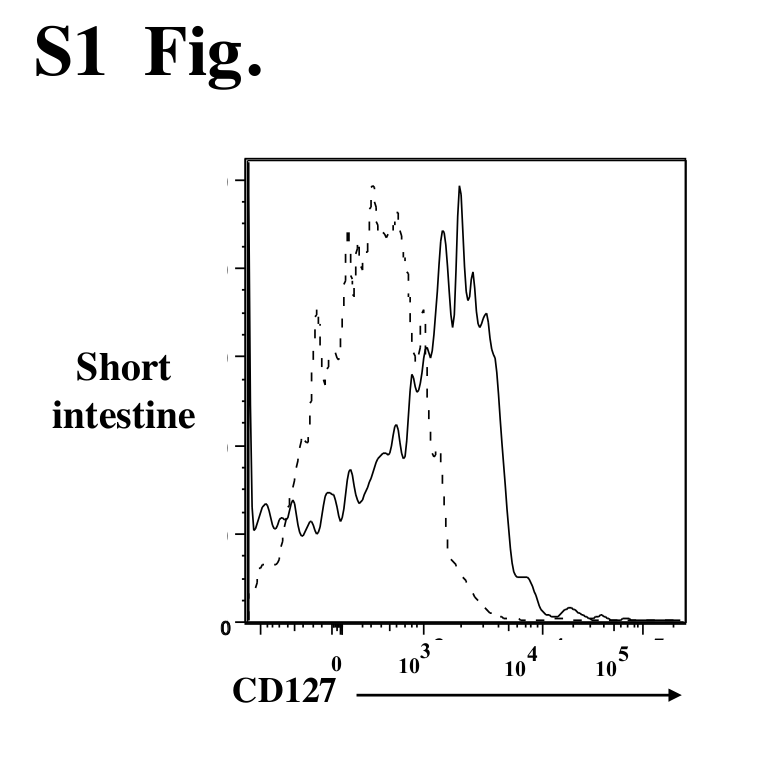

Supplement: S1 Fig — Flow cytometry of CD3−NKp46+ cells from lamina propria of short intestine stained with CD127. Data are representative of 4 mice. Continuous lines, specific antibodies; Dashed lines, isotype-matched control antibodies. (TIF) [file pone.0142920.s001.tif]

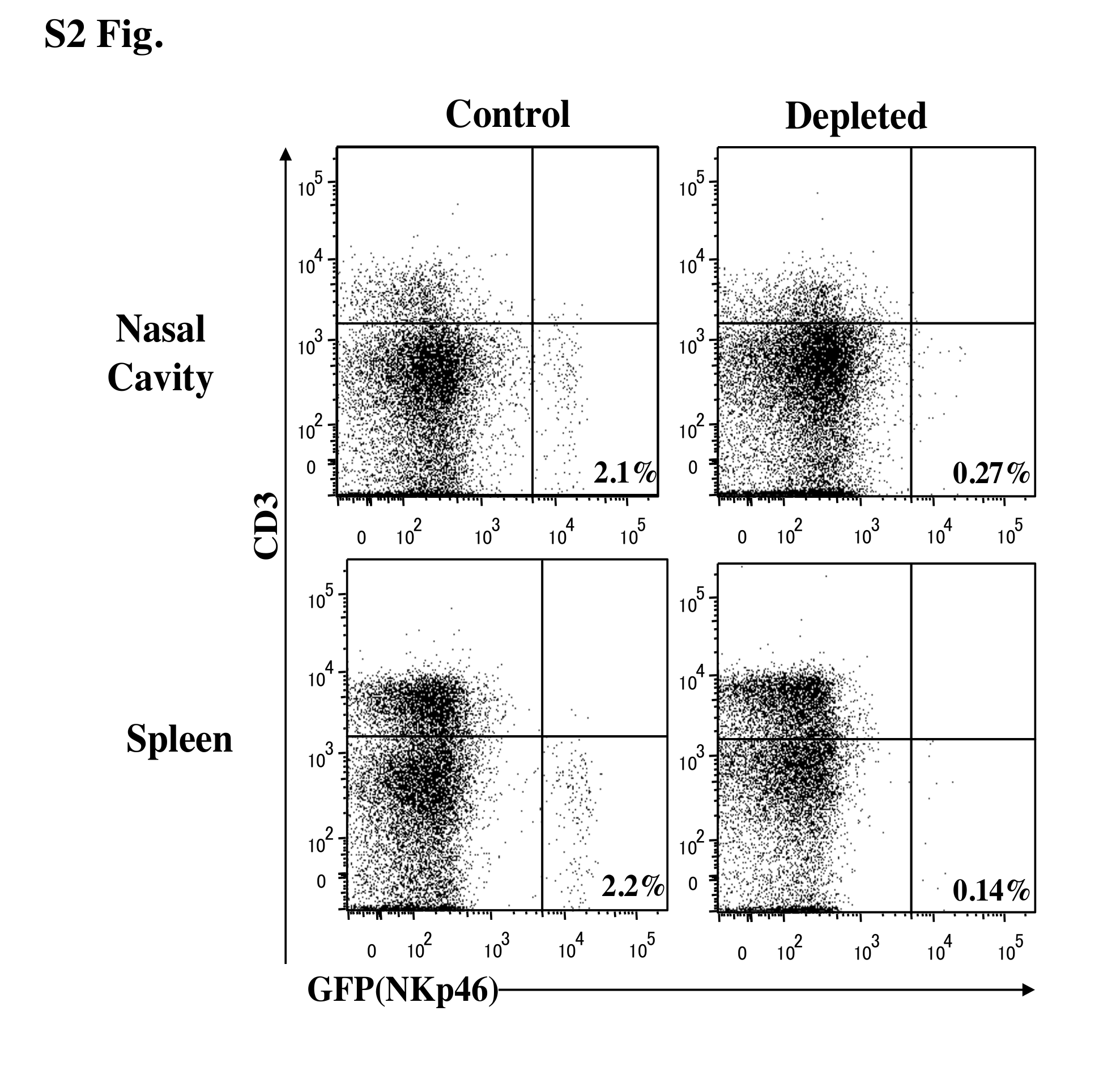

Supplement: S2 Fig — Depletion of nasal, splenic, and pulmonary NK cells. Lymphocytes of Ncr1 GFP/+ mice were analyzed 2 days after intraperitoneal injection of (right) 100 μg PK136 antibody or (left) isotype-matched control antibody. Horizontal axis, GFP (NKp46); vertical axis, NK1.1. Data are representative of 2 independent experiments using 4 mice each. (TIF) [file pone.0142920.s002.tif]
